# Brassicaceae-Based Biosolarization Reduces Lettuce *Fusarium* Wilt (FOLac) in Naturally Infested Soils

**DOI:** 10.3390/pathogens15080810

**Published:** 2026-08-01

**Authors:** Juan Manuel Arroyo, Lorenzo Badiali, Daniel Palmero

**Affiliations:** 1 Department of Agricultural Production, Escuela Técnica Superior de Ingeniería Agronómica, Alimentaria y de Biosistemas, Universidad Politécnica de Madrid, 28040 Madrid, Spain; juanmanuel.arroyo@upm.es; 2 Department of Agricultural and Environmental Sciences, University of Tuscia, 01100 Viterbo, Italy

**Keywords:** biosolarization, biofumigation, glucosinolates, *Fusarium oxysporum* f. sp. *lactucae*

## Abstract

Brassicaceae-based bio-disinfestation is a promising non-chemical option to manage lettuce *Fusarium* wilt caused by *Fusarium oxysporum* f. sp. *lactucae* (FOLac), but evidence integrating pathogen suppression, residue chemistry, and soil microbiome response remains limited. We conducted a greenhouse mesocosm assay with naturally infested soil to compare biofumigation (uncovered soil) and biosolarization (plastic-covered soil for 40 days) using five Brassicaceae residues (*Brassica carinata*, *B. juncea*, *B. napus*, *Sinapis alba* and *Raphanus sativus*) applied at two field-equivalent doses. Biosolarization created a distinct disinfestation environment, increasing soil temperature by an average of 2.74 °C and shifting oxidation–reduction potential toward reducing conditions. This strategy consistently reduced culturable *Fusarium* populations, *F. oxysporum*-assigned colony-forming units (CFU) and lettuce wilt severity compared with uncovered biofumigation. The strongest disease suppression was observed under plastic-covered conditions, including the soil-only control, indicating that the covered microenvironment was a major driver of suppressiveness, while Brassicaceae residues modulated the magnitude and consistency of the response. Glucosinolate profiling revealed contrasting residue chemistries among species, providing a biochemical context for interpreting species-dependent effects. Shotgun metagenomics further showed that biosolarization and biofumigation produced distinct genus-level microbial community structures, with significant effects of strategy, timepoint and their interaction, and higher Shannon diversity under biosolarization. Overall, the integration of disease severity, culture-based inoculum quantification, physicochemical indicators, residue chemistry and metagenomics supports Brassicaceae-based biosolarization as a promising pre-plant approach for suppressing FOLac wilt in naturally infested soils, with the plastic-covered disinfestation environment emerging as the main driver of suppression and Brassicaceae residues modulating the response.

## 1. Introduction

Soilborne diseases remain a major constraint in intensive horticultural systems, where short rotations and repeated cultivation favor progressive inoculum accumulation and recurrent outbreaks. Lettuce *Fusarium* wilt, caused by *Fusarium oxysporum* f. sp. *lactucae* (FOLac), is particularly difficult to manage because the pathogen persists in soil and crop residues, and management options after infestation are limited [1,2,3].

Since its first description in Japan, lettuce *Fusarium* wilt caused by *Fusarium oxysporum* f. sp. *lactucae* has diversified into several physiological races and expanded geographically [4,5,6,7,8]. The recent emergence and spread of race 4 in Europe and the Mediterranean region has increased concern in lettuce production systems [9,10,11,12,13,14]. Because resistance is often race-specific and curative options after soil infestation are limited, management relies largely on pre-plant decisions, especially cultivar selection, surveillance and soil interventions [2,3]. In North America, the discovery of novel pathogenic variants within race 1 isolates further illustrates the dynamic epidemiology of this pathosystem [15].

Among non-chemical options, Brassicaceae-based bio-disinfestation has gained attention because it integrates agronomic feasibility with biologically active chemistry. When Brassicaceae tissues are chopped and incorporated into moist soil, endogenous myrosinase can hydrolyse glucosinolates (GSLs), generating isothiocyanates (ITCs) and related sulfur volatiles with broad biocidal activity [16,17,18,19,20]. Species selection matters because GSL profiles differ substantially among Brassicaceae, while biomass production and residue handling influence bioactive release [19,20]. Previous work has commonly prioritized biofumigant selection using biomass and glucosinolate production as primary criteria [21]. However, biofumigation outcomes can be variable because efficacy depends on biofumigant species, amendment rate and soil moisture and temperature during decomposition.

Biosolarization combines organic amendments with plastic covering to intensify pathogen suppression through coupled thermal and amendment-driven effects, enhancing volatile retention and creating soil conditions that can be unfavorable to pathogens [22,23,24]. Evidence from other *Fusarium*-affected systems [21] supports the practical relevance of biosolarization as a single pre-plant intervention, while also illustrating that agronomic value depends on whether benefits persist into subsequent crop cycles [25,26]. From a microbiological perspective, biosolarization represents a combined chemical and physicochemical perturbation that can modify microbial community composition and activity. Here, we evaluate Brassicaceae-based bio-disinfestation strategies in a greenhouse mesocosm system using naturally infested soil, comparing biofumigation (residue incorporation without soil cover) versus biosolarization (residue incorporation followed by plastic cover). Five Brassicaceae species were tested at two field-equivalent amendment levels. We quantified disease severity in a susceptible lettuce cultivar and culture-based inoculum levels in soil, together with soil temperature and redox potential recorded during disinfestation. In addition, shotgun metagenomics at two timepoints (before and after treatment), was used to characterize microbiome shifts and short-term legacy effects. By integrating disease outcomes, inoculum reduction, and microbiome responses, this work aims to provide a robust basis for selecting Brassicaceae residues and bio-disinfestation strategy under realistic constraints where biosolarization is implemented once pre-plant.

## 2. Materials and Methods

### 2.1. Experimental Design and Soil

A greenhouse mesocosm experiment was conducted to evaluate Brassicaceae-based soil bio-disinfestation strategies against lettuce *Fusarium* wilt caused by *Fusarium oxysporum* f. sp. *lactucae* (FOLac). The study compared two disinfestation strategies: biofumigation (uncovered soil) and biosolarization (plastic-covered soil). Five Brassicaceae species were tested as green-residue amendments (Intersemillas S.A.U., Loriguilla, Valencia, Spain)) at two amendment levels (50% and 100% field-equivalent dose; Section 2.3), together with unamended controls. Pots were arranged in a randomized complete block design. Soil was collected from commercial horticultural fields with a history of lettuce *Fusarium* wilt (naturally infested soil). Overall, the experiment comprised two disinfestation strategies (biosolarization vs. biofumigation), two residue doses (50% and 100% field-equivalent), and five Brassicaceae species, with independent pots tracked as experimental units within blocks.

### 2.2. Pots, Experimental Units, and Controls

Cylindrical pots (25 cm diameter × 27 cm depth) were used as independent experimental units (pot surface area ≈ 0.05 m^2^) with one lettuce plant per pot. Treatments were assigned to pots in a randomized complete block design with two blocks. For each Strategy × Dose × Species combination, eight pots per block were established (*n* = 16 pots per combination across blocks) (i.e., *n* = 16 experimental units per treatment). Unamended controls (soil-only) were included under the corresponding physical conditions (plastic cover for biosolarization; no cover for biofumigation) and were replicated following the same blocking structure (*n* = 16 per control).

### 2.3. Brassicaceae Biomass Handling and Field-Equivalent Dose Definition

Brassicaceae biomass was harvested and incorporated as fresh chopped plant material (≈1–3 cm fragments). Although biomass was incorporated fresh, amendment rates were standardized and reported as field-equivalent dry-matter (DM) doses to ensure comparability among species. The 100% field-equivalent dose was based on previous field assays in which each Brassicaceae species was grown at the manufacturer-recommended sowing rate, and the total aboveground dry biomass produced per square meter was quantified [27]. Thus, the 100% dose represented the amount of aerial biomass that would realistically be harvested and incorporated into soil under recommended sowing conditions. The 50% dose was included to simulate a proportional reduction in sowing rate and, consequently, a proportional reduction in the amount of plant biomass incorporated. The 100% field-equivalent dose was calculated from species-specific total dry biomass (TDB; g DM m^−2^) measured at the end of the first productive cycle (27 March 2023; 186 DAS) and scaled to pot surface area (0.05 m^2^): Dose_100 (g DM pot^−1^) = TDB × 0.05; Dose_50 = 0.5 × Dose_100. Using published TDB values, Dose_100/Dose_50 were: *B. carinata* 30.25/15.13, *B. juncea* 40.62/20.31, *R. sativus* 35.85/17.92, and *S. alba* 27.00/13.50 g DM pot^−1^. *B. napus* doses were calculated analogously from its species-specific field biomass dataset.

### 2.4. Glucosinolate Analysis

Glucosinolate (GSL) profiles of Brassicaceae residues were determined following ISO 9167 with minor adaptations, and concentrations were reported as mean ± SD from eight biological replicates per Brassicaceae species (*n* = 8) [27]. Briefly, plant samples were frozen shortly after collection, lyophilized using a LyoQuest 55 Plus lyophilizer (Telstar, Terrassa, Spain), heat-treated to inactivate myrosinase, and ground with an IKA A11 basic analytical mill (IKA-Werke GmbH & Co. KG, Staufen, Germany). Glucosinolates were extracted, purified using DEAE-Sephadex A-25, and enzymatically desulfated. Glucotropaeolin was used as the internal standard (reference 89216; PhytoLab GmbH & Co. KG, Vestenbergsgreuth, Germany). Desulfoglucosinolates were analysed using a Waters HPLC system comprising a Waters 1525 binary pump, a Waters 2489 UV/visible detector, and a Waters 2707 autosampler (Waters Corporation, Milford, MA, USA), equipped with a Kinetex C18 column (250 × 4.6 mm, 100 Å, 5 µm; Phenomenex, Torrance, CA, USA). Concentrations are expressed as µmol g^−1^ dry weight (DW).

### 2.5. Biofumigation and Biosolarization Procedures

After residue incorporation, pots were irrigated to promote tissue hydrolysis and microbial activity. Biofumigation consisted of residue incorporation without plastic cover. Biosolarization consisted of residue incorporation followed by coverage with transparent polyethylene film (100 galgas) for 40 days (single pre-plant event); film edges were sealed to minimize gas exchange and enhance heat/volatile retention. Soil temperature and oxidation–reduction potential (ORP) were monitored during the disinfestation period as mechanistic indicators. ORP and temperature were measured using a combined pH/ORP/temperature meter (PCE-228, PCE Deutschland GmbH, Meschede, Germany), with ORP readings taken twice daily during a representative monitoring window. Soil temperature was recorded at 15 cm depth using dataloggers. After 40 days, films were removed and lettuce was transplanted.

### 2.6. Lettuce Cultivation and Disease Assessment

A susceptible lettuce cultivar ‘Maravilla de Verano’ (Semillas Batlle S.A. Sant Cugat del Vallès, Barcelona, Spain) was transplanted after the disinfestation period and grown for Cycle 1 under standard greenhouse practices. Vascular wilt severity was assessed at the end of Cycle 1 using a disease severity index (DSI; 0–5): 0 = healthy; 1 = very mild symptoms; 2 = mild yellowing; 3 = generalized yellowing and some wilting; 4 = severe wilting with marked vascular necrosis; 5 = collapsed/dead plant. Treatment-level DSI values were calculated as the mean of individual plant scores. Fresh weight and SPAD were recorded at harvest, SPAD-502^®^ chlorophyll meter (Konica Minolta Inc., Tokyo, Japan).

### 2.7. Culture-Based Inoculum Quantification and Isolate Identification

Soil samples were collected at defined assessment timepoints, air-dried at room temperature under aseptic conditions, homogenized, and sieved (200 μm). *Fusarium* inoculum was quantified by plating 0.02 g of sieved soil onto Komada selective medium [28], modified following Tello et al. [29]. Plates were incubated at 25 °C for 10 days under continuous fluorescent light, and colony-forming units (CFU) were recorded. Replicated plates were used per soil sample and summarized at pot level. Representative *Fusarium* colonies were purified on PDA and identified morphologically. A small subset of colonies recovered from modified Komada medium showing atypical morphology, i.e., morphology not consistent with *Fusarium* spp., was purified and subjected to Sanger sequencing of the ITS rDNA region to exclude misclassification. The ITS region was amplified using primers ITS1 (5′-TCCGTAGGTGAACCTGCGG-3′) and ITS4 (5′-TCCTCCGCTTATTGATATGC-3′), following White et al. [30]. PCR reactions were performed in a final volume of 25 µL containing template DNA, PCR buffer, MgCl_2_, dNTPs, primers and DNA polymerase. The amplification profile consisted of an initial denaturation at 95 °C for 4 min 30 s; 40 cycles of denaturation at 95 °C for 30 s, annealing at 50 °C for 30 s and extension at 72 °C for 1 min; and a final extension at 72 °C for 3 min. Amplicons were checked by agarose gel electrophoresis and sent for Sanger sequencing. Three representative sequences, corresponding to three morphotypes initially represented by five colonies including replicates, were compared against the NCBI nucleotide collection (nt) database using the BLASTn web interface (https://blast.ncbi.nlm.nih.gov/Blast.cgi, accessed on 27 July 2026). These sequences were not used for population-level inference, but only as a quality-control step to confirm that atypical colonies were not *Fusarium*. The sequences were deposited in GenBank under accession numbers PZ528630–PZ528632. The culture collection was preserved at −80 °C in 20% (*v*/*v*) glycerol stocks at Universidad Politécnica de Madrid.

### 2.8. Shotgun Metagenomics: Sampling, Sequencing, and Bioinformatics

Shotgun metagenomic sequencing was used to characterize soil microbiome shifts at two timepoints. For clarity, metagenomic sampling timepoints were defined as follows: T0 corresponds to before treatment (provider suffix “b”), and T1 corresponds to after treatment (provider suffix “a”). Accordingly, samples labeled as bc and ac represented unamended soil-only controls at T0 and T1, respectively. At each timepoint and for each disinfestation strategy (biosolarization vs. biofumigation), soil was sampled from each Brassicaceae treatment (five species) with three biological replicates per species (*n* = 3), resulting in 15 Brassicaceae-amended samples per Strategy × Timepoint combination. In addition, soil-only controls were sequenced with three biological replicates per Strategy × Timepoint (*n* = 3), yielding 18 samples per Strategy × Timepoint and 72 metagenomic libraries overall (2 strategies × 2 timepoints × 18 samples).

Shotgun metagenomic data were processed by the sequencing service provider according to its standard metagenomic workflow. Briefly, raw reads were quality-filtered using fastp [31], screened for potential host-derived reads, assembled de novo using MEGAHIT [32], and processed for gene prediction and non-redundant gene catalog construction. Taxonomic annotation was performed by aligning unigenes against the Micro_NR database using DIAMOND-based searches [33]. Functional annotation against KEGG, eggNOG and CAZy databases was also generated by the provider [34,35,36]; however, because the present study was designed primarily to evaluate disease suppression, inoculum reduction and treatment-associated microbial community shifts, the manuscript focuses on taxonomic community structure, alpha diversity and global microbiome responses. Detailed pathway-level interpretation was considered outside the main scope of the present work.

### 2.9. Statistical Analysis

For mesocosm outcomes, disease severity (DSI), SPAD, fresh weight and CFU were analyzed using models appropriate to the response distribution. CFU counts were log-transformed when required (e.g., log10(CFU + 1)). For the culture-based inoculum data, total *Fusarium* CFU and *F. oxysporum*-assigned CFU were analyzed using non-parametric Kruskal–Wallis tests. Strategy effects were evaluated within each dose by comparing biofumigation and biosolarization. In addition, within-panel comparisons among Brassicaceae species and the soil-only control were performed separately for each Strategy × Dose combination. For the plant-response variables, DSI, SPAD and fresh weight were also analyzed using Kruskal–Wallis tests within each dose, comparing Brassicaceae-amended biofumigation and biosolarization treatments. Significance was assessed at α = 0.05.

For metagenomics, hypothesis testing was simplified to two factors: Strategy (biosolarization vs. biofumigation) and Timepoint (T0 vs. T1). Beta diversity was evaluated using Bray–Curtis dissimilarity computed from genus-level relative abundances and visualized by PCoA. PERMANOVA (Bray–Curtis, genus level), following the non-parametric multivariate analysis of variance framework of Anderson [37], was used to test the effects of Strategy, Timepoint, and their interaction, with Amendment (Brassicaceae vs. soil-only control) included as an additional factor. PERMANOVA R^2^ values were interpreted as multivariate effect-size estimates, representing the proportion of Bray–Curtis variation explained by each factor. For disease severity, inoculum and plant-response variables, treatment effects were interpreted using the combination of statistical significance, post hoc group separation and graphical dispersion of biological replicates.

## 3. Results

### 3.1. Soil Physicochemical Conditions During Bio-Disinfestation

**Figure 1:**
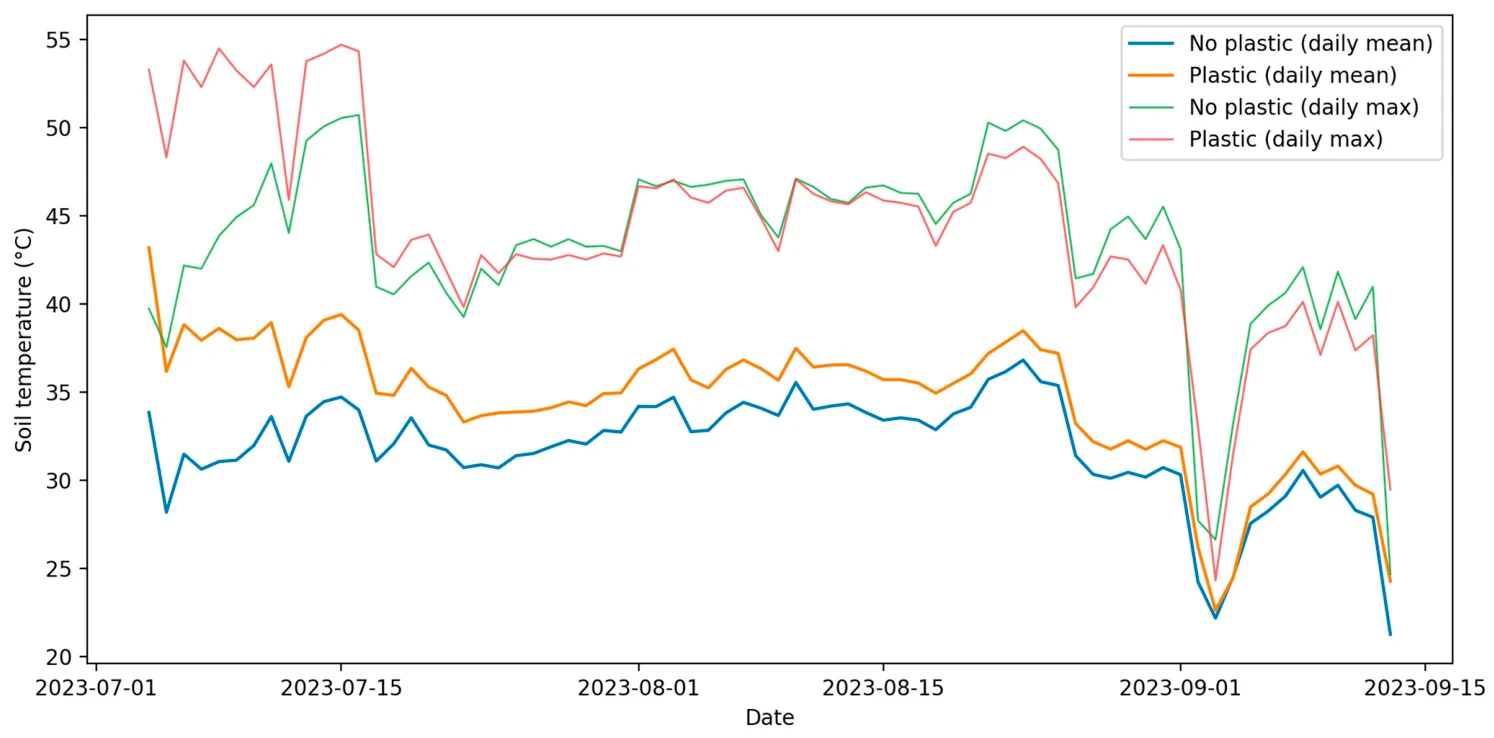
Soil temperature under biosolarization (plastic cover) and biofumigation (no cover) during the disinfestation period (4 July–13 September 2023). Over the overlapping monitoring window (1706 hourly pairs), plastic cover increased soil temperature by an average of +2.74 °C (plastic−no plastic), with higher daily maxima under plastic (up to 54.3 °C) than without plastic (up to 50.5 °C).

Plastic-covered pots (biosolarization) developed a distinct physicochemical environment compared with uncovered pots (biofumigation). Mean redox potential (ORP) was −68.1 mV in covered treatments and +42.4 mV in uncovered treatments. Soil temperatures during the disinfestation period were higher under plastic cover than without cover (Figure 1).

### 3.2. Glucosinolate Profiles of Brassicaceae Residues

Glucosinolate composition differed among Brassicaceae residues (Table 1). *Brassica juncea* and *B. carinata* displayed low-diversity profiles dominated by sinigrin (SIN). *Sinapis alba* showed high aromatic glucosinolates, with sinalbin (SIB) as a major component, together with substantial glucobrassicanapin (GBN). *Raphanus sativus* showed a mixed profile dominated by glucoerucin (ERU) and other aliphatic glucosinolates, with detectable indolic glucobrassicin (GBC). The full glucosinolate dataset is provided in Table S1 in Supplementary File S1.

### 3.3. Culture-Based Inoculum Reduction After Treatments

**Figure 2:**
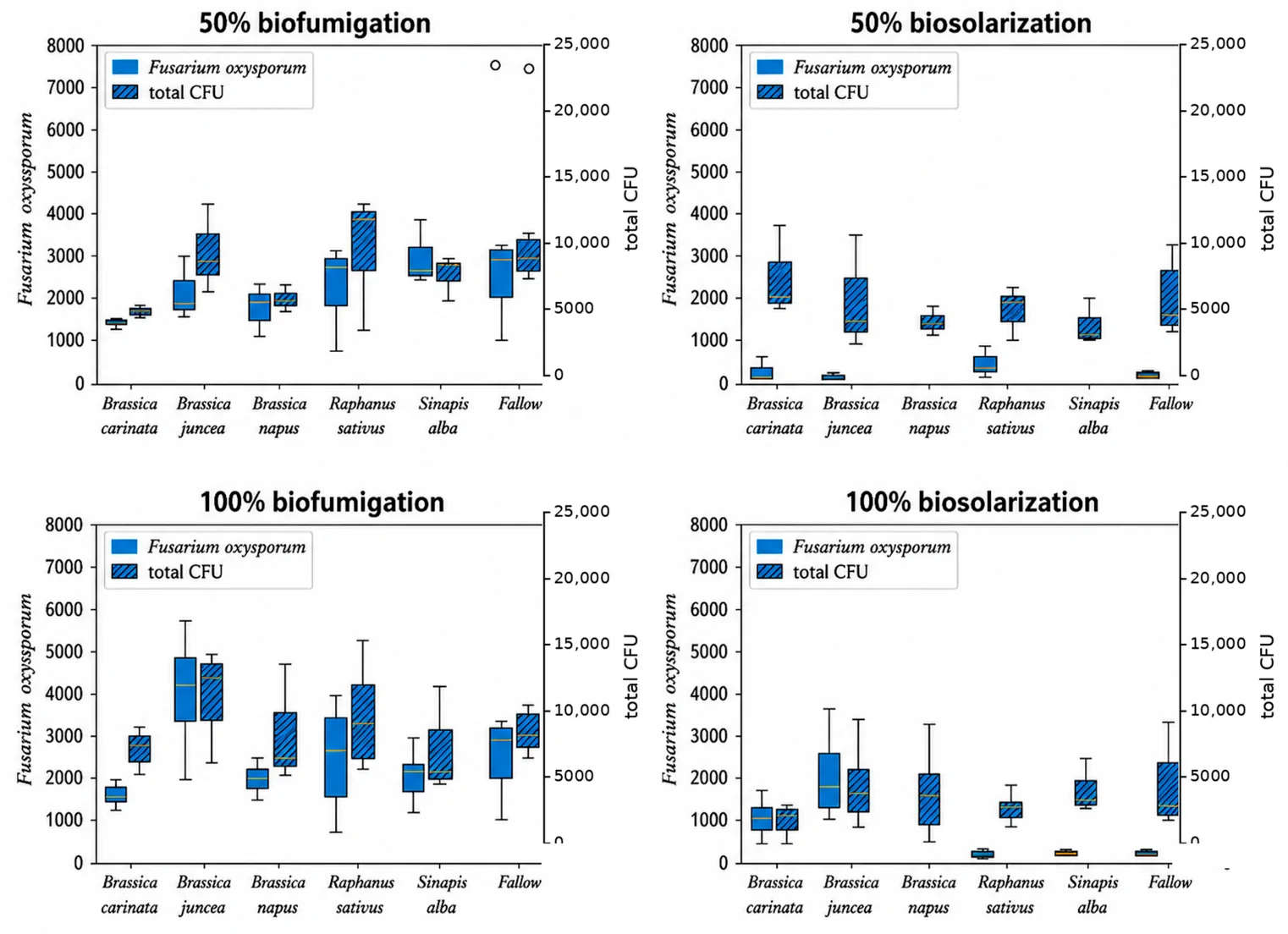
Effect of Brassicaceae-based bio-disinfestation on cultivable *Fusarium* populations. Boxplots show total *Fusarium* CFU (hatched boxes; right y-axis) and *F. oxysporum*-assigned CFU (plain boxes; left y-axis) quantified on Komada selective medium. Panels correspond to 50% biofumigation, 50% biosolarization, 100% biofumigation, and 100% biosolarization; within each panel, results are displayed by Brassicaceae species and the unamended control (Fallow).

Selective plating on Komada medium indicated that the bio-disinfestation treatments reduced cultivable *Fusarium* populations relative to the untreated control. Across Brassicaceae species and amendment doses, biosolarization generally produced lower CFU values than biofumigation, both for total *Fusarium* CFU and for *F. oxysporum*-assigned CFU (Figure 2). To verify that *Fusarium* counts on Komada medium were not biased by morphologically atypical colonies, representative non-typical morphotypes were Sanger-sequenced (ITS) and consistently matched non-*Fusarium* taxa (e.g., Ectophoma/Didymella-like lineages). This supports that CFU values reported as total *Fusarium* and *F. oxysporum*-assigned CFU were not inflated by morphologically ambiguous non-*Fusarium* colonies. Residue-associated trends were observed within some Strategy × Dose combinations, although these effects were less consistent than the overall difference between biosolarization and biofumigation. Accordingly, the CFU data did not support a single universal ranking of Brassicaceae species in terms of inoculum suppression. Instead, residue-specific responses were interpreted as treatment-dependent effects occurring within the broader suppressive environment generated by biosolarization.

Non-parametric comparisons confirmed that the main statistically supported pattern was the reduction in culturable inoculum under biosolarization relative to biofumigation. When strategies were compared within each dose, total *Fusarium* CFU were significantly lower under biosolarization at both 50% dose (Kruskal–Wallis, H = 9.04, *p* = 0.0026) and 100% dose (H = 15.70, *p* < 0.001). This effect was even stronger for *F. oxysporum* CFU, which were markedly reduced under biosolarization at both 50% dose (H = 31.56, *p* < 0.001) and 100% dose (H = 31.72, *p* < 0.001). By contrast, within each Strategy × Dose panel, differences among Brassicaceae species and the soil-only control were weaker and mostly non-significant, except for *F. oxysporum* CFU under 100% biosolarization (H = 11.54, *p* = 0.042). These results support that the disinfestation strategy was the dominant driver of culturable inoculum reduction, while residue-specific effects were more variable and treatment-dependent. Accordingly, the CFU data did not support a single universal ranking of Brassicaceae species in terms of inoculum suppression. Instead, residue-specific responses were interpreted as treatment-dependent effects occurring within the broader suppressive environment generated by biosolarization.

### 3.4. Fusarium Wilt Suppression and Lettuce Plant Status (DSI, SPAD, and Fresh Weight)

**Figure 3:**
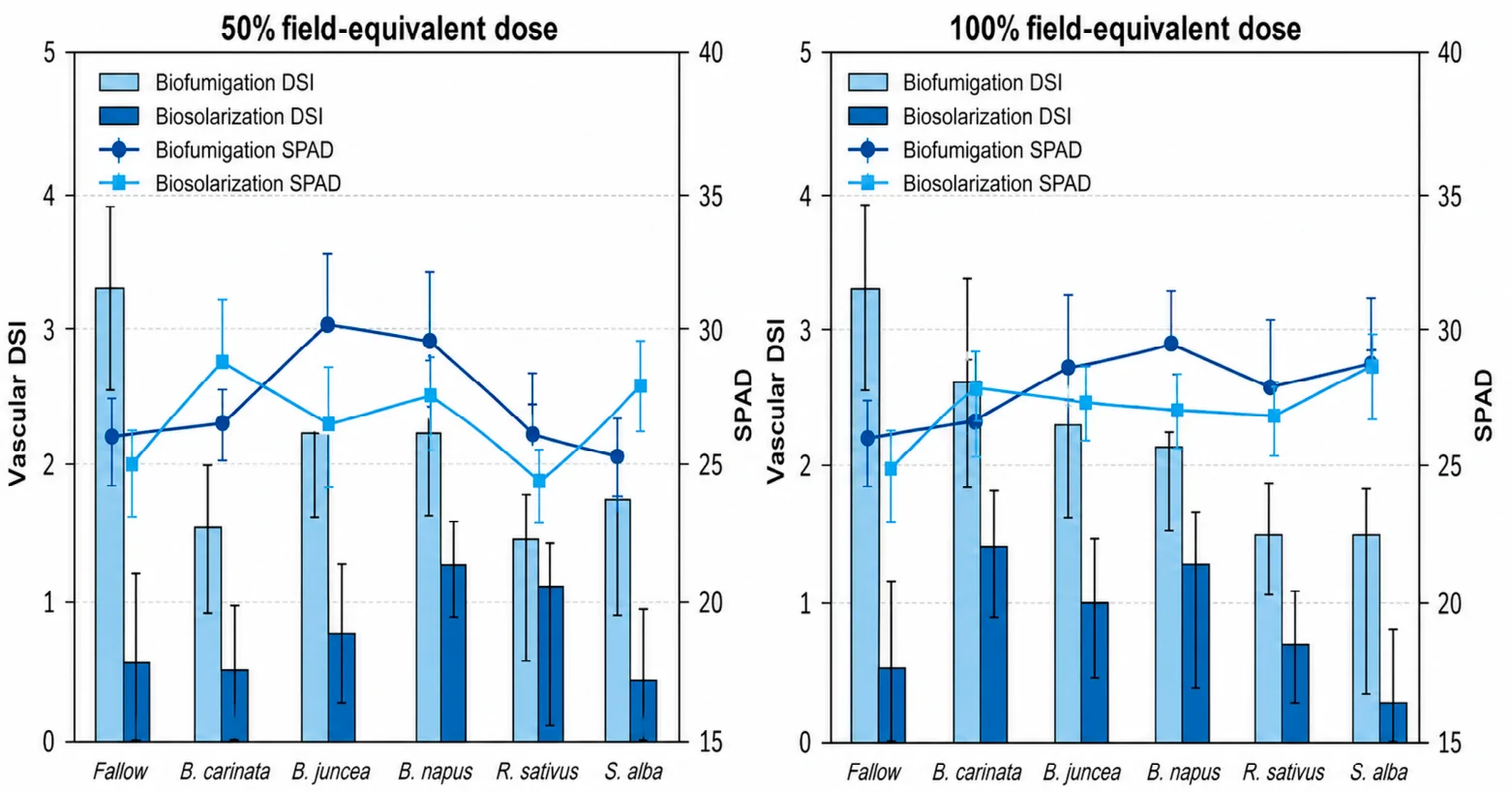
Effect of biofumigation and biosolarization on lettuce *Fusarium* wilt severity and leaf greenness. Bars show mean vascular disease severity index (DSI; left y-axis) ± SD for each biofumigant species at 50% and 100% field-equivalent dose under biofumigation (no plastic cover) and biosolarization (plastic cover). Lines show mean SPAD values (right y-axis) ± SD for the corresponding treatment groups.

Lettuce vascular wilt severity differed among disinfestation strategies, amendment doses, and Brassicaceae residues (Figure 3). Overall, biosolarization markedly reduced DSI relative to biofumigation across biofumigant species and at both amendment doses. The fallow control under biofumigation showed high disease severity (DSI = 3.33 at both doses), whereas the corresponding soil-only control under biosolarization showed low symptom expression (DSI = 0.50), indicating strong suppression under plastic-covered conditions even in the absence of Brassicaceae residues. Within biosolarization treatments, DSI values were generally close to the minimum of the scale; for example, at 100% dose, *S. alba* and *R. sativus* showed particularly low DSI (0.25 and 0.71, respectively), whereas *B. napus* showed higher residual symptom expression (≈1.33). SPAD values varied within a narrower range than DSI (Figure 3) and showed only modest separation among treatments.

Fresh weight showed high within-treatment variability and substantial overlap among treatments. Across Brassicaceae-amended pots, mean fresh weight under biofumigation was 49.5 ± 18.6 g (50% dose; *n* = 32) and 51.8 ± 16.0 g (100% dose; *n* = 31), while biosolarization averaged 76.1 ± 16.6 g (50% dose; *n* = 32) and 58.2 ± 13.8 g (100% dose; *n* = 31). Despite differences in group means—more pronounced at 50% dose—the wide dispersion and overlapping ranges indicate that biomass accumulation at the assessment time was a less consistent endpoint than vascular DSI for capturing treatment effects in this pathosystem.

Non-parametric comparisons supported the stronger reduction in vascular wilt severity under biosolarization. When Brassicaceae-amended treatments were compared within each dose, DSI values were significantly lower under biosolarization than under biofumigation at both 50% dose (Kruskal–Wallis, H = 18.72, *p* < 0.001) and 100% dose (H = 19.58, *p* < 0.001). Fresh weight was also higher under biosolarization, with a stronger effect at 50% dose (H = 25.85, *p* < 0.001) and a weaker but significant effect at 100% dose (H = 4.12, *p* = 0.042). By contrast, SPAD values did not differ significantly between strategies at 50% dose (H = 0.61, *p* = 0.436) and showed only a marginal trend at 100% dose (H = 3.43, *p* = 0.064). These results confirm that vascular DSI was the most consistent plant-response endpoint for detecting treatment effects in this pathosystem.

### 3.5. Association Between F. oxysporum Abundance and Vascular Disease Severity

**Figure 4:**
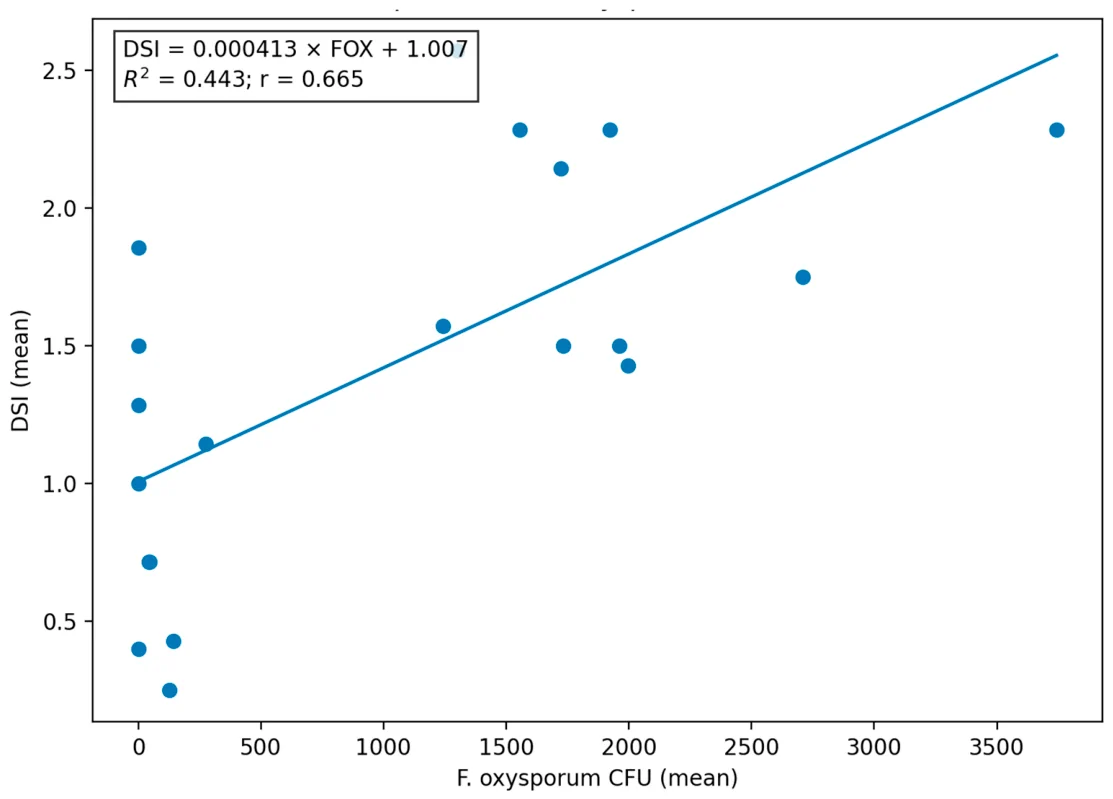
Relationship between *Fusarium oxysporum* abundance and vascular disease severity. Scatter plot showing the association between mean *F. oxysporum*-assigned CFU in soil and mean vascular disease severity index (DSI) in lettuce across treatments. The fitted linear regression line is shown.

When treatment means were considered, *F. oxysporum* abundance was positively associated with vascular disease severity (Figure 4). Linear regression indicated a moderate positive relationship (R^2^ = 0.443; r = 0.665).

### 3.6. Shotgun Metagenomics: Community Structure, Alpha Diversity, and PERMANOVA

**Figure 5:**
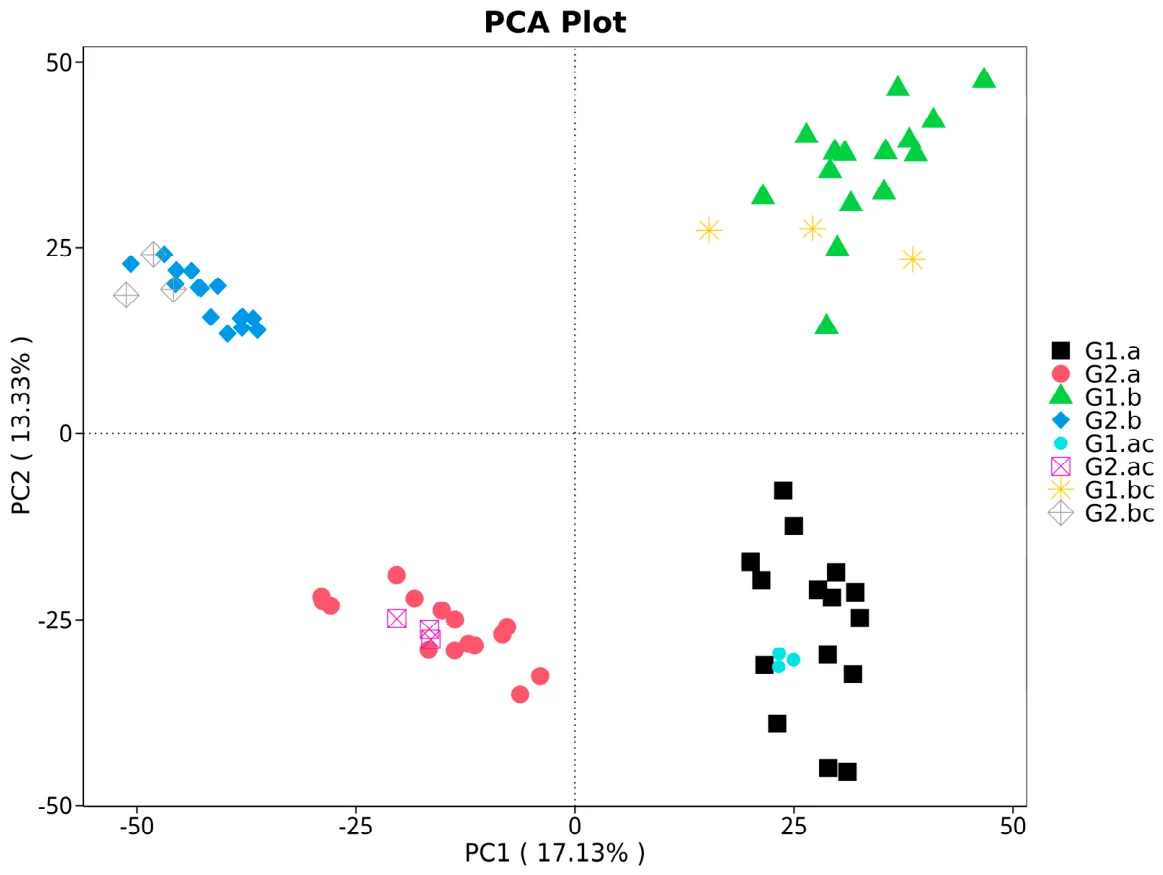
Genus-level beta diversity (Bray–Curtis PCoA). Samples are labeled according to the provider coding scheme: G1 = biosolarization, G2 = biofumigation; a = after treatment, b = before treatment; ac/bc denote soil-only controls (no Brassicaceae residues) under the corresponding physical conditions.

For clarity, metagenomic sampling timepoints are defined as follows: T0 corresponds to “before treatment” (provider suffix “b”), and T1 corresponds to “after treatment” (provider suffix “a”). Genus-level beta diversity (Bray–Curtis PCoA) showed a structured distribution of samples according to disinfestation strategy and timepoint (Figure 5). The first two axes explained 17.13% and 13.33% of the variation, respectively. However, pre-treatment samples assigned to the biosolarization (G1.b) and biofumigation (G2.b) groups were already separated to some extent, indicating baseline heterogeneity among mesocosm soil samples before treatment application. Therefore, the ordination was interpreted cautiously, with emphasis on the overall Strategy × Timepoint structure rather than on pre-treatment separation alone. Brassicaceae residues did not produce a clear species-specific clustering pattern in the global PCoA, suggesting that residue effects on community structure were weaker than the broader effects associated with disinfestation strategy and sampling timepoint. Control samples without Brassicaceae residues (ac/bc) also showed separation between T0 and T1, consistent with a measurable effect of the physical disinfestation conditions. A complementary species-level PCoA showed a broadly consistent ordination pattern and is provided as Supplementary Figure S1.

**Figure 6:**
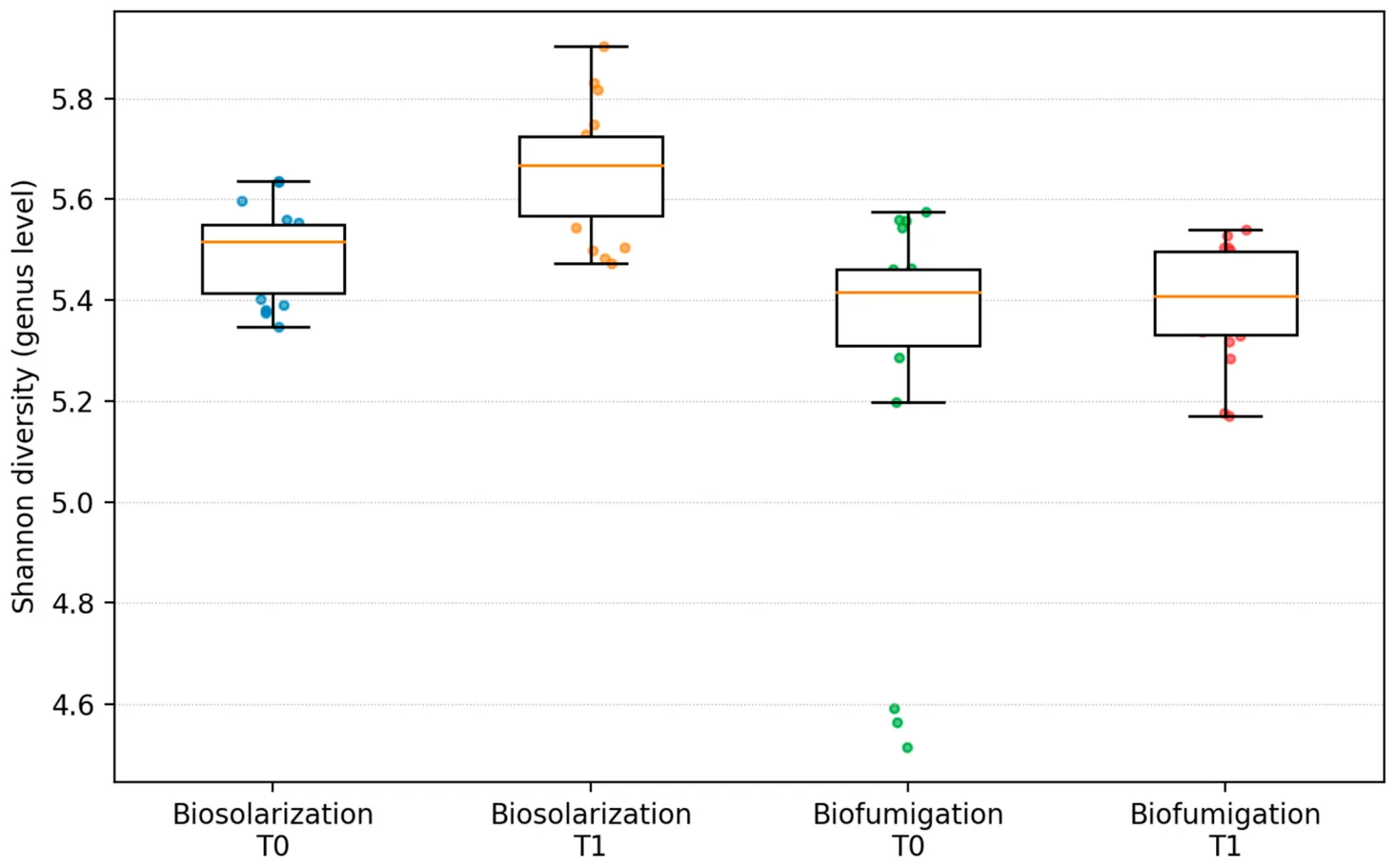
Genus-level alpha diversity by strategy and timepoint. Boxplots show Shannon diversity (genus level) for biosolarization (G1) and biofumigation (G2) at T0 (before; “b”) and T1 (after; “a”). Center lines represent medians; boxes indicate the interquartile range; whiskers extend to 1.5× IQR; points represent individual samples. Group sizes were *n* = 15 per strategy × timepoint.

PERMANOVA confirmed that strategy, timepoint and their interaction contributed significantly to genus-level community structure, supporting the interpretation that the treatments were associated with measurable shifts in soil microbial composition over time. Genus-level alpha diversity (Shannon index) differed between strategies and timepoints (Figure 6; Table S4 in Supplementary File S1). PERMANOVA on Bray–Curtis distances (genus level) confirmed significant effects of Strategy and Timepoint, as well as a significant Strategy × Timepoint interaction (Table 2). In contrast, the aggregated factor Amendment (Brassicaceae residues vs. soil-only controls) showed a weaker and non-significant effect at α = 0.05.

Together, these ordination and diversity patterns indicate that the treatments were associated not only with changes in overall community structure, but also with biologically interpretable shifts in microbial groups potentially involved in organic matter turnover, competitive soil colonization and treatment-driven microbial succession.

## 4. Discussion

Our results show that biosolarization (plastic-covered soil; G1) provided stronger suppression of lettuce *Fusarium* wilt than uncovered biofumigation (G2). This was reflected in consistent reductions in culture-based inoculum, including Komada colony-forming unit (CFU) counts and *F. oxysporum*-assigned CFU after treatment, together with lower disease severity index (DSI) values in lettuce. The contrast between strategies is consistent with the established efficacy of soil solarization as a hydrothermal disinfestation approach [22,23]. It also agrees with evidence that combining solar heating with organic amendments can further enhance pathogen suppression (“biosolarization”) [21,24,25,26]. Importantly, the soil-only control under plastic (ac/bc) also showed low symptom expression compared with the uncovered soil-only control. This indicates that the physical environment created under plastic can be a major driver of suppressiveness, while Brassicaceae residues likely modulate the magnitude and consistency of suppression across species and doses. This interpretation is consistent with previous studies indicating that bio-disinfestation efficacy depends on the interaction between organic amendment chemistry, soil temperature, water availability, gas diffusion and microbial activity, rather than on a single suppressive mechanism [18,21,24,25,26].

Plastic cover increased soil temperature and shifted soil redox conditions toward more reducing values (ORP −68.1 mV under cover vs. +42.4 mV uncovered). These physicochemical conditions are coherent with the mechanistic basis of solarization/biosolarization and related organic-amendment-based disinfestation approaches, where heat accumulation, restricted gas exchange and microbial oxygen consumption may contribute to the suppression of soilborne pathogens [21,22,23,24,25,26]. In these treatments, heat accumulation, restricted gas exchange and elevated microbial oxygen demand can create environments less favorable for soilborne pathogens. They can also alter competitive interactions within the soil microbiome. The strong suppression observed in soil-only plastic controls supports the interpretation that the covered microenvironment (heat plus reduced oxygen availability) can itself contribute substantially to disease suppression, providing a mechanistic foundation on which residue effects may add further perturbation.

Brassicaceae residues showed contrasting glucosinolate (GSL) profiles (Table 1, Table S1), including SIN-dominated profiles (*B. juncea* and *B. carinata*), ERU-rich profiles (*R. sativus*), and mixed aromatic/aliphatic patterns (*S. alba*). These differences are relevant because biofumigation efficacy may depend on GSL hydrolysis and on the formation, retention and degradation of isothiocyanates (ITCs) and other sulfur volatiles in soil [17,18,19,20]. Previous studies have shown that the antimicrobial potential of Brassicaceae residues is strongly linked to glucosinolate concentration, tissue disruption, myrosinase activity and the release and persistence of ITCs in soil, although the final suppressive effect is highly context-dependent [17,18,19,20,27]. However, ITCs and other bioactive degradation products were not quantified directly in soil in this study. Therefore, residue chemistry should be interpreted as a biochemical context and plausible explanatory layer rather than direct proof of causality or as evidence of a direct correlation between in-soil chemical dynamics and disease suppression. This limitation is particularly relevant because ITCs are highly reactive and transient compounds in soil, making their reliable quantification under bio-disinfestation conditions analytically challenging. Future studies should consider quantifying bioactive degradation products, particularly isothiocyanates and other sulfur-containing volatiles, directly in soil and to strengthen the mechanistic link between Brassicaceae residue chemistry, microbial community shifts and disease suppression.

Although Brassicaceae species differed markedly in glucosinolate composition, species-dependent effects on disease suppression and culturable inoculum were less consistent than the overall effect of disinfestation strategy. Under biosolarization, disease severity was generally low across most residues, with *S. alba* and *R. sativus* showing particularly low DSI values in some treatments, whereas *B. napus* showed comparatively higher residual symptom expression. However, CFU-based differences among Brassicaceae species were mostly weaker than the contrast between biosolarization and biofumigation. Therefore, the present results do not support a single universal ranking of biofumigant species. Rather, they suggest that residue chemistry modulated the suppressive response within a broader physicochemical environment driven by plastic cover. From a practical perspective, alternating or combining Brassicaceae residues with contrasting glucosinolate profiles may be preferable to relying on a single species. Such diversification may help reduce the risk of selecting soil microbial communities specifically adapted to rapidly degrade a narrow set of glucosinolate-derived compounds, potentially maintaining the persistence and activity of isothiocyanates and related bioactive molecules in soil. Thus, residue selection should consider not only short-term disease suppression, but also the functional diversity of glucosinolate profiles and their possible implications for long-term soil microbial adaptation.

Shotgun metagenomics indicated that both the disinfestation environment and sampling time relative to treatment were associated with measurable changes in microbiome structure. Bray–Curtis PCoA at genus level showed a structured distribution between biosolarization (G1) and biofumigation (G2), and between T0 (before treatment; provider suffix “b”) and T1 (after treatment; provider suffix “a”) samples (Figure 5). This pattern agrees with previous evidence that solarization, biosolarization and organic-amendment-based soil disinfestation can modify rhizosphere or soil microbial communities, including studies reporting changes in microbial composition during solarization or biosolarization treatments [24,25,26]. Soil-only controls without Brassicaceae residues (bc/ac) followed the same temporal structure. This supports the idea that the physical treatment environment alone can drive community shifts even in the absence of plant amendments.

Alpha diversity patterns were coherent with these multivariate results. Shannon diversity at genus level was higher under biosolarization than under biofumigation at both timepoints, and differed between T0 and T1 (Figure 6; Table S4 in Supplementary File S1). Together, these patterns indicate a structured reconfiguration of soil communities rather than a simple “sterilization” effect. This interpretation is consistent with biosolarization studies showing that pathogen suppression may be accompanied by shifts in the resident soil microbial community rather than by a complete elimination of microbial populations [24,25,26]. PERMANOVA (Bray–Curtis, genus level) corroborated the ordination patterns, showing significant effects of Timepoint (T0 vs. T1) and Strategy (G1 vs. G2), and a significant Strategy × Timepoint interaction (Table 2). By contrast, the aggregated factor Amendment (Brassicaceae vs. soil-only controls) showed a weaker global effect. This suggests that the covered vs. uncovered environment was a dominant driver of whole-community structure, while residue effects may have been expressed more subtly through specific taxa or functions.

From an ecological perspective, these microbial shifts suggest that bio-disinfestation did not act only as a direct physical or chemical stress, but also promoted a reorganization of the soil microbial community. Differential abundance analyses comparing Brassicaceae-amended soils with their corresponding soil-only controls after treatment showed strategy-specific microbial signatures. Under biosolarization, Brassicaceae-amended soils were associated with groups such as Actinomycetota, Chloroflexota/Anaerolineae, Aggregatilineaceae/*Aggregatilinea*, Longimicrobiaceae/Longimicrobium, Iamia and Capillimicrobium. Under biofumigation, differential taxa included Longimicrobiaceae/*Longimicrobium*, *Chitinophagia*, *Microvirga*, *Humisphaera*, *Lysobacter*, *Metabacillus*, *Brevundimonas*, *Gaiella* and Chloroflexota-related groups. Several of these taxa are consistent with soil microbial functions such as organic matter turnover, adaptation to fluctuating physicochemical conditions, competitive colonization or potential antagonistic activity in residue-amended and biosolarized soils [24,25,26]. Such shifts may contribute indirectly to pathogen suppression by modifying resource availability, niche occupation and microbial interactions after treatment. However, the present study evaluated community-level patterns rather than experimentally confirming the suppressive role of individual taxa. Therefore, these microbial groups should be interpreted as candidates associated with bio-disinfestation-driven community reorganization, rather than as confirmed causal agents of FOLac suppression.

A key operational feature of biosolarization is that it is typically implemented once pre-plant. The strong suppression observed here under realistic constraints, namely a single intervention and naturally infested soil, supports biosolarization as a practical option for lettuce production systems. Repeated disinfestation events are often costly in time, space and labor. The consistent reshaping of the microbiome between T0 and T1 further suggests that the intervention induces a measurable ecological shift. This shift may contribute to short-term legacy effects after treatment and cropping. Where possible, future validation should quantify persistence of suppression across subsequent crop cycles and across soil types to better define operational windows of efficacy.

This was a greenhouse mesocosm study on a naturally infested soil, which increases applied relevance but also implies that soil history and background microbiota may influence outcomes. Metagenomics was evaluated at two timepoints and interpretation focused on global community structure and diversity; direct measurements of in-soil ITCs/volatiles were not performed. Future work should (i) validate these findings across soil types and production settings; (ii) quantify bioactive degradation products, particularly isothiocyanates and other sulfur-containing volatiles, directly in soil; and (iii) integrate functional metagenomic pathway analysis to better link Brassicaceae residue chemistry, microbial functional potential and disease suppression.

Brassicaceae-based biosolarization should be considered within the broader context of non-chemical soilborne disease management strategies, including organic amendments, anaerobic soil disinfestation, composts, microbial biocontrol agents, biofertilizers and plant-derived bioactive compounds. Compared with approaches based on the introduction of selected beneficial microorganisms or commercial biostimulant formulations, biosolarization acts through a combination of mechanisms, including heat accumulation, oxygen depletion, residue decomposition, release of bioactive compounds and subsequent shifts in soil microbial community structure. This multifactorial mode of action may be advantageous in complex soilborne pathosystems, where pathogen suppression rarely depends on a single mechanism.

Beyond FOLac wilt, Brassicaceae-based bio-disinfestation may have wider relevance within integrated soil health strategies, particularly where chemical fumigation is restricted or undesirable. Biofumigation and biosolarization have been explored against diverse soilborne fungi, oomycetes, bacteria, plant-parasitic nematodes and, more recently, highly persistent plant viruses associated with infected crop residues. However, efficacy is strongly target-dependent and is influenced by residue chemistry, amendment dose, soil moisture, temperature and exposure time. In this regard, the recent failure of biofumigation and biosolarization to inactivate tomato brown rugose fruit virus (ToBRFV) and cucumber green mottle mosaic virus (CGMMV) in soil and substrates highlights that not all pathogen groups respond similarly to these treatments [38]. Therefore, pathogen-specific sensitivity, inoculum distribution and local agronomic conditions will determine whether the level of suppression observed for FOLac can be reproduced in other pathosystems.

Although the present study provides controlled evidence that Brassicaceae-based biosolarization can reduce FOLac inoculum and lettuce wilt severity, its field-scale performance will depend strongly on environmental boundary conditions. The experiment was conducted in a greenhouse mesocosm using a single naturally infested soil; therefore, extrapolation to commercial fields should be made with caution. Soil texture may influence treatment efficacy by modifying water retention, aeration, heat transfer and the diffusion or retention of bioactive compounds released during residue decomposition. Consequently, the magnitude of disease suppression observed here should be interpreted as evidence of potential efficacy under controlled conditions. Field validation across contrasting soil textures and seasonal weather scenarios will be required to define the robustness and scalability of the approach.

## 5. Conclusions

Brassicaceae-based biosolarization (plastic-covered soil) was associated with marked suppression of lettuce *Fusarium* wilt caused by *Fusarium oxysporum* f. sp. *lactucae* in naturally infested soil under greenhouse mesocosm conditions. In Cycle 1, disease severity (DSI) was consistently lower under biosolarization than under uncovered biofumigation, in parallel with reduced cultivable inoculum on Komada selective medium (total *Fusarium* and *F. oxysporum*-assigned CFU). The physicochemical conditions recorded during disinfestation—higher soil temperatures under plastic and more reducing redox potential—are consistent with a mechanistic contribution of the covered microenvironment to suppression, while the contrasting glucosinolate profiles among Brassicaceae residues provide a biochemical context to interpret species-dependent variation without implying direct causality in the absence of in-soil isothiocyanate measurements. Shotgun metagenomics further supported that the intervention reshaped the soil microbiome, with significant strategy- and time-dependent differences in genus-level community composition and diversity. Overall, integrating disease, inoculum, mechanistic indicators and metagenomics supports biosolarization as a strong non-chemical option to reduce reliance on chemical fumigants in intensive lettuce systems, and highlights priorities for future validation across soils and environments and for linking residue chemistry to in-soil bioactive dynamics.

## Figures and Tables

**Table 1:** Glucosinolate composition of Brassicaceae residues used for bio-disinfestation. Values are expressed as µmol g^−1^ dry weight (DW) and reported as mean ± SD (*n* = 8). Abbreviations: SIN, sinigrin; ERU, glucoerucin; GBN, glucobrassicanapin; SIB, sinalbin; RAA, glucoraphanin; GBC, glucobrassicin; EPI, epiprogoitrin; GNA, gluconapin. Total glucosinolates (TOT) corresponds to the sum of quantified compounds.

Species	n	Total (µmol g^−1^ DW)	SIN	ERU	GBN	SIB	RAA	GBC	EPI	GNA
*B. carinata*	8	8.92 ± 0.53 ^d^	8.79 ± 0.52 ^b^	0.00 ± 0.00 ^b^	0.00 ± 0.00 ^c^	0.00 ± 0.00 ^b^	0.00 ± 0.00 ^b^	0.08 ± 0.01 ^b^	0.00 ± 0.00 ^c^	0.00 ± 0.00 ^d^
*B. juncea*	8	40.01 ± 3.81 ^a^	38.98 ± 3.78 ^a^	0.00 ± 0.00 ^b^	0.00 ± 0.00 ^c^	0.00 ± 0.00 ^b^	0.00 ± 0.00 ^b^	0.12 ± 0.01 ^b^	0.00 ± 0.00 ^c^	0.20 ± 0.03 ^c^
*B. napus*	8	5.87 ± 0.25 ^e^	0.00 ± 0.00 ^c^	0.00 ± 0.00 ^b^	2.50 ± 0.12 ^b^	0.00 ± 0.00 ^b^	0.14 ± 0.05 ^b^	0.05 ± 0.00 ^b^	0.00 ± 0.00 ^c^	1.04 ± 0.05 ^a^
*R. sativus*	8	28.19 ± 1.89 ^b^	0.00 ± 0.00 ^c^	15.13 ± 1.46 ^a^	0.00 ± 0.00 ^c^	0.00 ± 0.00 ^b^	9.13 ± 2.53 ^a^	3.18 ± 0.13 ^a^	0.48 ± 0.10 ^b^	0.00 ± 0.00 ^d^
*S. alba*	8	23.62 ± 0.86 ^c^	0.00 ± 0.00 ^c^	0.00 ± 0.00 ^b^	10.70 ± 0.28 ^a^	10.70 ± 0.52 ^a^	0.02 ± 0.02 ^b^	0.01 ± 0.01 ^c^	1.81 ± 0.07 ^a^	0.31 ± 0.07 ^b^

Different superscript letters within each column indicate significant differences among Brassicaceae species according to Tukey’s HSD test (α = 0.05).

**Table 2:** PERMANOVA (Bray–Curtis; genus level) testing effects of Strategy (biosolarization vs. biofumigation), Timepoint (T0 vs. T1), their interaction, and Amendment (Brassicaceae vs. control).

Factor	R^2^	F	*p*-Value
Strategy	0.112	12.906	0.001
Timepoint	0.259	30.002	0.001
Strategy × Timepoint	0.041	4.777	0.002
Amendment	0.018	2.058	0.071

R^2^ values indicate the proportion of Bray–Curtis dissimilarity explained by each factor and were used as multivariate effect-size estimates.

## Data Availability

The datasets supporting this study are openly available in e-cienciaDatos: Biofumigant Species Evaluation (Proy. OPTIMSOIL), Version 3, DOI: 10.21950/0UHHSU. Raw shotgun metagenomic reads are publicly available in the NCBI Sequence Read Archive (SRA) under BioProject accession number PRJNA1478125. Sample-specific BioSample and SRA Run accession IDs are provided in Table S2 of Supplementary File S1.

## Supplementary Materials

The following supporting information can be downloaded at: https://www.mdpi.com/article/10.3390/pathogens15080810/s1, Supplementary File S1: Supplementary tables provided in a single Excel file with separate worksheets: Table S1, full glucosinolate profile of Brassicaceae residues; Table S2 in Supplementary File S1, shotgun metagenomics QC statistics per sample; Table S3 in Supplementary File S1, assembly statistics per sample (scaftigs ≥ 500 bp); Table S4 in Supplementary File S1, gene catalog summary statistics; Table S5 in Supplementary File S1, alpha diversity indices at genus level by Strategy × Timepoint. Supplementary Figure S1: Species-level beta diversity based on Bray–Curtis principal coordinates analysis (PCoA) of soil metagenomic samples. Samples are labelled according to the provider coding scheme: G1 = biosolarization, G2 = biofumigation; a = after treatment, b = before treatment; ac/bc denote soil-only controls without Brassicaceae residues.

## Abbreviations

The following abbreviations are used in this manuscript:

CFU	colony-forming units
DSI	disease severity index (vascular)
DW	dry weight
FOLac	*Fusarium oxysporum* f. sp. *lactucae*
GSL	glucosinolate(s)
HPLC	high-performance liquid chromatography
ORP	oxidation–reduction potential
PCoA	principal coordinates analysis
PERMANOVA	permutational multivariate analysis of variance
SPAD	chlorophyll meter index (Soil–Plant Analysis Development)
