# Supplementary material for: Brassicaceae-Based Biosolarization Reduces Lettuce Fusarium Wilt (FOLac) in Naturally Infested Soils
Source: Pathogens. 2026 Aug 1;15(8):810. doi: 10.3390/pathogens15080810 (PMC13516823; doi:10.3390/pathogens15080810)

Figure S1. Species-level beta diversity based on Bray–Curtis principal coordinates analysis (PCoA) of soil metagenomic samples. Samples are labelled according to the provider coding scheme: G1 = biosolarization, G2 = biofumigation; a = after treatment, b = before treatment; ac/bc denote soil-only controls without Brassicaceae residues.

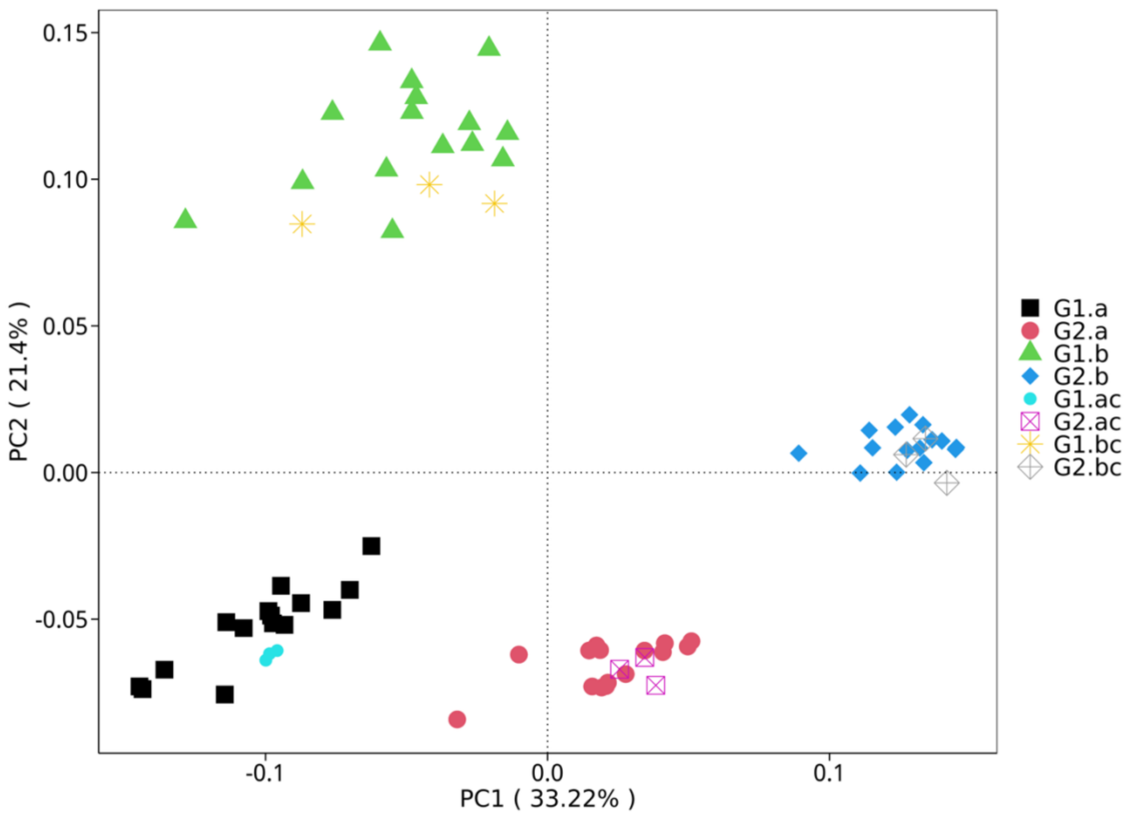

Supplement: Supplementary file 1 [file pathogens-15-00810-s001.zip › Supplementary_Figure_S1_species_level_PCoA.pdf]
